# Activity and regulation by growth factors of calmodulin-dependent protein kinase III (elongation factor 2-kinase) in human breast cancer[Fn tlnote1]

**DOI:** 10.1038/sj.bjc.6690012

**Published:** 1999-09-24

**Authors:** T G Parmer, M D Ward, E J Yurkow, V H Vyas, T J Kearney, W N Hait

**Affiliations:** Departments of Pharmacology, Internal Medicine, Surgery and The Cancer Institute of New Jersey, University of Medicine and Dentistry of New Jersey, New Brunswick, NJ, 08901, USA; Department of Pharmacology and Toxicology, Rutgers University, Piscataway, NJ, 08855, USA

**Keywords:** Ca^2+^/calmodulin-dependent protein kinase III (elongation factor-2 kinase), breast cancer, breast cancer mitogens, cell cycle, protein synthesis

## Abstract

Calmodulin-dependent protein kinase III (CaM kinase III, elongation factor-2 kinase) is a unique member of the Ca^2+^/CaM-dependent protein kinase family. Activation of CaM kinase III leads to the selective phosphorylation of elongation factor 2 (eEF-2) and transient inhibition of protein synthesis. Recent cloning and sequencing of CaM kinase III revealed that this enzyme represents a new superfamily of protein kinases. The activity of CaM kinase III is selectively activated in proliferating cells; inhibition of the kinase blocked cells in G_0_/G_1_-S and decreased viability. To determine the significance of CaM kinase III in breast cancer, we measured the activity of the kinase in human breast cancer cell lines as well as in fresh surgical specimens. The specific activity of CaM kinase III in human breast cancer cell lines was equal to or greater than that seen in a variety of cell lines with similar rates of proliferation. The specific activity of CaM kinase III was markedly increased in human breast tumour specimens compared with that of normal adjacent breast tissue. The activity of this enzyme was regulated by breast cancer mitogens. In serum-deprived MDA-MB-231 cells, the combination of insulin-like growth factor I (IGF-I) and epidermal growth factor (EGF) stimulated cell proliferation and activated CaM kinase III to activities observed in the presence of 10% serum. Inhibition of enzyme activity blocked cell proliferation induced by growth factors. In MCF-7 cells separated by fluorescence-activated cell sorting, CaM kinase III was increased in S-phase over that of other phases of the cell cycle. In summary, the activity of Ca^2+^/CaM-dependent protein kinase III is controlled by breast cancer mitogens and appears to be constitutively activated in human breast cancer. These results suggest that CaM kinase III may contribute an important link between growth factor/receptor interactions, protein synthesis and the induction of cellular proliferation in human breast cancer. © 1999 Cancer Research Campaign


*British Journal of Cancer* (1999) **79**(1), 59–64
© 1999 Cancer Research Campaign

### Activity and regulation by growth factors of calmodulin-dependent protein kinase III (elongation factor 2-kinase) in human breast cancer*

**TG Parmer[1], MD Ward[1], EJ Yurkow[2], VH Vyas[1], TJ Kearney[1] and WN Hait[1]**

[1]Departments of Pharmacology, Internal Medicine, Surgery and The Cancer Institute of New Jersey, University of Medicine and Dentistry of New Jersey, Robert Wood Johnson Medical School, New Brunswick, NJ 08901, USA; and [2]Department of Pharmacology and Toxicology, Rutgers University, Piscataway, NJ 08855, USA

**Summary** Calmodulin-dependent protein kinase III (CaM kinase III, elongation factor-2 kinase) is a unique member of the $Ca^{2+}$/CaM-dependent protein kinase family. Activation of CaM kinase III leads to the selective phosphorylation of elongation factor 2 (eEF-2) and transient inhibition of protein synthesis. Recent cloning and sequencing of CaM kinase III revealed that this enzyme represents a new superfamily of protein kinases. The activity of CaM kinase III is selectively activated in proliferating cells; inhibition of the kinase blocked cells in $G_0$/$G_1$-S and decreased viability. To determine the significance of CaM kinase III in breast cancer, we measured the activity of the kinase in human breast cancer cell lines as well as in fresh surgical specimens. The specific activity of CaM kinase III in human breast cancer cell lines was equal to or greater than that seen in a variety of cell lines with similar rates of proliferation. The specific activity of CaM kinase III was markedly increased in human breast tumour specimens compared with that of normal adjacent breast tissue. The activity of this enzyme was regulated by breast cancer mitogens. In serum-deprived MDA-MB-231 cells, the combination of insulin-like growth factor I (IGF-I) and epidermal growth factor (EGF) stimulated cell proliferation and activated CaM kinase III to activities observed in the presence of 10% serum. Inhibition of enzyme activity blocked cell proliferation induced by growth factors. In MCF-7 cells separated by fluorescence-activated cell sorting, CaM kinase III was increased in S-phase over that of other phases of the cell cycle. In summary, the activity of $Ca^{2+}$/CaM-dependent protein kinase III is controlled by breast cancer mitogens and appears to be constitutively activated in human breast cancer. These results suggest that CaM kinase III may contribute an important link between growth factor/receptor interactions, protein synthesis and the induction of cellular proliferation in human breast cancer.

**Keywords:** $Ca^{2+}$/calmodulin-dependent protein kinase III (elongation factor-2 kinase); breast cancer; breast cancer mitogens; cell cycle; protein synthesis

The response of breast cancer cells to growth factors requires a complex interaction between growth factors and growth factor receptors, followed by the activation of well-defined signal transduction pathways that mediate a proliferative response. Not only must signal transduction pathways be activated, but cellular checkpoints must also be inactivated for a cell to proliferate. Thus, the proliferative response includes the activation of early-response genes as well as the inactivation of cell cycle repressor genes (Muller et al, 1984), and may require the coordinated activation and suppression of protein synthesis (Makino et al, 1984; Greenberg et al, 1986).

There has been considerable interest in developing diagnostic, prognostic and therapeutic modalities based on this information. Although much attention has focused on growth factor/receptor interactions, such as the immediate downstream effector elements seen in receptor-activated tyrosine kinases, less attention has been given to signalling events further downstream. In this regard, calmodulin (CaM), a ubiquitous mediator of calcium-dependent signal transduction, represents a compelling area for investigation into the proliferative responses of malignant cells (Hait and Lazo, 1986; Lu and Means, 1993). For example, Chafouleas et al (1981) reported that CaM was required for re-entry of quiescent Chinese hamster ovary (CHO) cells into the cell cycle. Rasmussen and Means (1987) demonstrated that overexpression of CaM increased cell proliferation by decreasing the $G_1$/S transit time, and that CaM antisense blocked this effect (Rasmussen and Means, 1989). Although CaM is a constitutively expressed protein in non-dividing cells (Veigl et al, 1984), cellular transformation to malignancy has been associated with increases in intracellular CaM (Chafouleas et al, 1981). In addition, a positive correlation between CaM levels and the growth rate of malignant cells has been demonstrated (MacManus et al, 1981).

Despite these observations, the mechanism(s) by which CaM exerts its effects on the growth of malignant cells remains unclear. CaM-dependent protein kinases are central to many of the functions mediated by CaM. Calmodulin-dependent protein kinase III (CaM kinase III, elongation factor-2 kinase) is a unique member of the CaM-dependent protein kinase family with one known substrate, elongation factor 2 (eEF-2). The recently reported purification, sequencing and cloning of CaM kinase III (Hait et al,

*Received 26 August 1997*
*Revised 16 February 1998*
*Accepted 24 March 1998*

*Correspondence to:* WN Hait, The Cancer Institute of New Jersey, UMDNJ, Robert Wood Johnson Medical School, 195 Little Albany Street, New Brunswick, NJ 08901, USA

*This work was supported by Grants CA 43888 and CA 57142 from the National Cancer Institute, and generous contribution from the Hyde and Watson Foundation.

1996; Redpath et al, 1996; Ryazanov et al, 1997) revealed that the enzyme lacks homology to other threonine/serine or tyrosine kinases. The recognition that one other enzyme, myosin heavy-chain kinase A, shares sequence homology with CaM kinase III in the catalytic domain (Futey et al, 1995; Ryazanov et al, 1997) suggests that CaM kinase III represents a new superfamily of protein kinases (Ryazanov et al, 1997).

The activity of CaM kinase III is selectively activated in proliferating cells (Bagaglio and Hait, 1994; Cheng et al, 1995; Hait et al, 1996; Parmer et al, 1997). Rottlerin, a drug that inhibits CaM kinase III activity, blocks cells at the $G_0/G_1$-S boundary and induces cell death (Parmer et al, 1997).

Despite the emerging information on CaM kinase III in the control of cell proliferation and viability, nothing is known about the regulation of the enzyme in human breast cancer. Therefore, we studied the activity of CaM kinase III and its regulation by mitogens and expression during the cell cycle in several breast cancer models.

### MATERIALS AND METHODS

### Breast tumour specimens and cell lines

MCF-7 (an oestrogen-dependent human breast carcinoma cell line), MDA-MB-231 (an oestrogen-independent human breast carcinoma cell line), HL-60 (human promyelocytic leukaemia cells), T98G (human glioblastoma cells), C6 (*N*-nitrosomethylurea-induced rat glioma line), OVCAR-3 (human ovarian adenocarcinoma cells) and KB (human oral epidermoid carcinoma) cells were all obtained from the American Type Culture Collection. MCF-7 cells were grown in Iscove's modified Eagle medium (IMEM) and MDA-MB-231 cells in Leibovitz (L-15) media. HL-60 were grown in RPMI-1640, T98G were grown in Ham's F-10 Dulbecco's modified Eagle medium (DMEM) (1:10), and C6, KB and OVCAR-3 cells were grown in DMEM. All media were obtained from Gibco-BRL, Grand Island, NY, USA. All cell lines were supplemented with 10% fetal bovine serum (FBS), 100 U $ml^{-1}$ penicillin and 100 μg $ml^{-1}$ streptomycin. All cell lines had doubling times of approximately 24 h. Cultures were maintained at 37°C in a humidified atmosphere containing 5% carbon dioxide/95% air. In experiments designed to elucidate the role of CaM kinase III in the mitogenic response, cells in logarithmic phase of growth were seeded at a density of $1 \times 10^5$ cells $ml^{-1}$ and were grown in a serum-free chemically defined medium (MCDB 105). All cells were routinely checked and found to be free of mycoplasma and fungi.

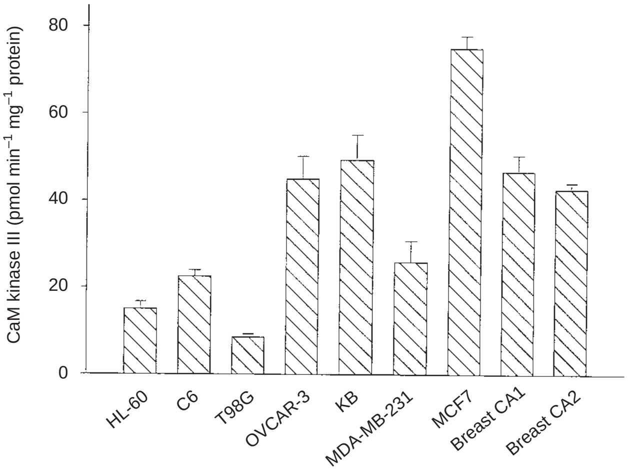

**Figure 1** CaM kinase III activity in malignant cell lines and in human breast carcinoma. CaM kinase III activity was determined by measuring the phosphorylation of eEF-2. Cell lysates from HL-60 leukaemia cells, C6 (*N*-nitrosomethylurea-induced rat glioma line), T98G (human glioblastoma cells), OVCAR-3 (human ovarian adenocarcinoma cells), KB (human oral epidermoid carcinoma), MDA-MB-231 (oestrogen receptor-negative breast cancer), MCF-7 (an oestrogen-dependent human breast carcinoma cell line) and human breast carcinoma (CA 1 and CA 2) were phosphorylated in assay mixtures as described in Materials and methods. Equal amounts of protein were incubated in the presence of 1.5 mM calcium chloride and 2 μM CaM. Purified eEF-2 was added as exogenous substrate. After SDS-PAGE by autoradiography, the phosphorylated eEF-2 band was quantified with an Ambis radioanalytical imaging system. Each bar represents the mean ± s.e.m. of at least three experiments

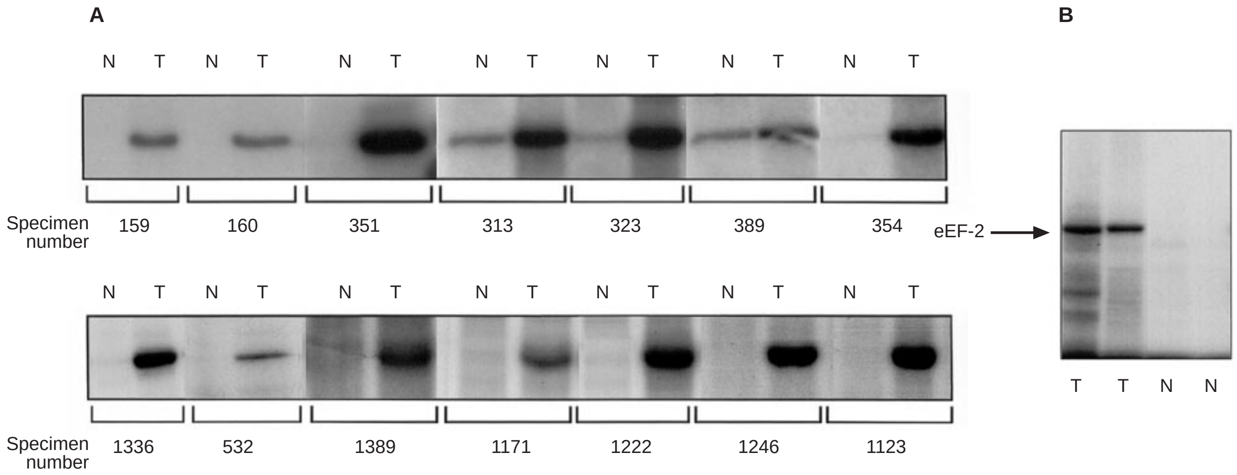

**Figure 2** Increased activity of CaM kinase III in human breast cancer specimens. (**A**) Unselected human breast cancer (T) and adjacent normal breast tissue (N) specimens were obtained at the time of surgery by the Tissue Retrieval Service of The Cancer Institute of New Jersey. Tissues were prepared and assayed for CaM kinase III activity as described in Materials and methods. Numbers at the bottom of each set refer to specimen identification in the Tissue Retrieval Service databank. Evaluation of 'normal' breast tissue from specimens 323 and 389 found evidence of tumour cells (see Results and discussion). (**B**) Unselected human breast cancers (T) and normal mammary tissue taken from patients undergoing breast reduction surgery (N) were homogenized and analysed as described in Figure 1

© Cancer Research Campaign 1999

Sixteen fresh human breast cancer specimens (14 with matched adjacent normal tissue) were obtained at the time of initial excisional biopsy or at the time of definitive surgery. In addition, normal mammary tissue was obtained during two breast reduction mammoplasty operations. All specimens were procured through the Tissue Retrieval Service of The Cancer Institute of New Jersey, were frozen in liquid nitrogen within 1 h of excision and stored at – 80°C until processed. No patients had received preoperative hormonal treatment or chemotherapy.

### Activity of CaM kinase III

Cell monolayers were removed using 0.05% trypsin, 0.5 mM EDTA (Gibco-BRL) and washed in phosphate-buffered saline (PBS). Fresh-frozen breast samples and cells were homogenized in ice-cold buffer containing 25 mM Hepes pH 7.4, 100 mM sodium chloride, 20 mM sodium pyrophosphate, 2 mM EDTA, 0.5 mM phenylmethylsulphonyl fluoride (PMSF), 1.25 μg $ml^{-1}$ leupeptin, 1.25 μg $ml^{-1}$ pepstatin A and 2.5 μg $ml^{-1}$ soybean trypsin inhibitor using a Polytron homogenizer. The homogenate was then centrifuged at 15 000 ***g*** for 30 min at 4°C. The protein concentration of the supernatants was determined according to the method of Bradford using a Bio-Rad protein assay kit (Bio-Rad Laboratories, Richmond, CA, USA) with bovine serum albumin (BSA) as the standard. The phosphorylation of eEF-2 was measured in 40-μl reaction mixtures containing 50 mM Hepes pH 7.6, 10 mM magnesium chloride, 10 μg of protein, 1.5 mM calcium chloride, 0.5 μg eEF-2 and 2 μM CaM (Calbiochem, San Diego, CA, USA). After the addition of 20 μM [$\gamma$-$^{32}$P]ATP (3 μCi per assay; Amersham, UK), reactions were carried out at 30°C for 2 min. Reactions were linear with respect to time of incubation and amount of tissue. Phosphorylation was terminated by the addition of 20 μl of 3 × Laemmli sample buffer containing 190 mM Tris (pH 6.8), 6% sodium dodecyl sulphate (SDS), 30% glycerol, 15% 2-mercaptoethanol and 0.003% bromophenol blue dye. Samples were boiled for 5 min and proteins were separated by SDS-PAGE according to the method of Laemmli using an 8% resolving gel and 4% stacking gel. Gels were fixed and stained in 40% methanol/10% acetic acid/0.1% Coomassie brilliant blue R-250 (Bio-Rad), destained and dried.

For the analysis of radiolabelled proteins, Kodak X-Omat XRP-5 film was exposed to dried gels at – 70°C for 24–48 h. Phosphoimagery was performed using an Ambis radioanalytical imagery system (Ambis, San Diego, CA, USA) and specific activity was calculated from background-corrected data.

### Sorting of MCF-7 cells

Approximately 5 × $10^4$ MCF-7 cells were stained with Hoechst 33342 (10 μg $ml^{-1}$) in growth medium for 30 min before harvesting. The cells were removed from the flasks by trypsinization, centrifuged (1000 ***g***) for 5 min at 4°C, and the pellet was washed and resuspended in PBS containing Hoechst dye. Before analysis, the stained cells were passed through a 26-gauge needle to ensure a single-cell suspension. Cells were analysed and sorted using an Epics Elite flow cytometer (Coulter Electronics, Hialeah, FL, USA) equipped with a Coherent 90, 5 W, adjustable wavelength laser. Linear peak and integrated blue fluorescence signals were collected using a 380-nm laser block, 588-nm dichroic long and 418-nm longpass filters. A blue (peak signal) parameter histogram was used to exclude cell debris, doublets and clumps from the sorted populations. In a typical experiment, with sort speeds of 2000 total events $s^{-1}$, purity of the sorted populations was greater than 98%. The histograms were analysed using Epics Cyto-Logic Software.

### Analysis of responses to growth factors

Growth factor studies were carried out using cells in logarithmic phase plated initially in serum-supplemented media as described above. After a 4–6 h attachment period, cells were washed three times with serum-free MCDB 105 medium and cultured for 24 h without mitogens to obtain baseline measurements.

Cells were then exposed, for 24 h, to either 10% FBS, insulin-like growth factor I (IGF-I) (20 ng $ml^{-1}$) and/or epidermal growth factor (EGF) (10 ng $ml^{-1}$) and/or rottlerin (5 μM), an inhibitor of CaM kinase III activity (Carl Roth, Karlsruhe, Germany). Control cells were maintained in MCDB 105 without growth factors or serum.

Cell proliferation was measured by BrdU incorporation. Briefly, MDA-MB-231 cells were incubated with 10 μM BrdU for 45 min at 37°C and harvested by trypsinization. Cells were then washed with ice-cold PBS, resuspended in 200 μl PBS and fixed by dropwise addition of cold 70% ethanol while vortexing. The cells were resuspended and incubated for 30 min in 2 N hydrochloric acid/0.5% Triton X-100 in PBS and neutralized by rinsing once in 0.1 M sodium tetraborate (pH 8.5). Fluorescein isothiocyanate (FITC)-conjugated anti-BrdU antibodies (Becton Dickinson) were added (10 μg per sample) in 50 μl of 0.5% Tween 20/1% BSA in PBS and incubated for 30 min. The cells were washed and resuspended in 1 ml of PBS containing 5 μg $ml^{-1}$ propidium iodide. Fluorescence intensities were determined by quantitative flow cytometry and profiles were generated on a Becton Dickinson FACScan analyser.

### Statistical analysis

Statistical analysis of results from the cell line experiments was carried out using ANOVA with a Student–Newman–Keuls method of multiple comparisons (Snedecor and Cochran, 1980). Comparisons of breast cancer surgical specimens and adjacent normal tissue were performed with the Wilcoxon rank-sum test.

### RESULTS AND DISCUSSION

CaM kinase III is a unique mitogen-activated protein kinase, confirmed by purification (Hait et al, 1996) and cloning of the full-length cDNAs from rat (Redpath et al, 1996; Ryazanov et al, 1997), *Caenorhabditis elegans*, mouse and human (Ryazanov et al, 1997). We found that the sequence of the kinase was distinct from all known protein kinases with the exception of myosin heavy-chain kinase A and, therefore, appears to represent a new superfamily of protein kinases (Ryazanov et al, 1997).

The current data demonstrate that the activity of CaM kinase III is markedly increased in human breast cancer, and that the activity of this enzyme is controlled by growth factor stimulation. As shown in Figure 1, the activity of CaM kinase III in human breast cancer cell lines was equal to or greater than that seen in a variety of cell lines with similar rates of proliferation. In fact, MCF-7 cells had the greatest specific activity of the enzyme found in an analysis of seven cell lines and several human breast tumours.

The activity of the kinase is also markedly increased in breast cancer specimens compared with that of adjacent normal breast

© Cancer Research Campaign 1999

tissue (Figure 2A). Fourteen specimens were examined from ten 'lumpectomies' and four mastectomies. In adjacent 'normal' tissue, we found either no detectable activity (specimens 159, 160, 351, 354, 532, 1123, 1171, 1222, 1246, 1336 and 1389) or far less activity (specimens 313, 323 and 389). Pathological evaluation of adjacent 'normal' breast tissue from two of these specimens (323 and 389) found evidence of tumour cells. Because no activity was detected in 'normal' breast tissue samples taken from patients undergoing reduction mammoplasty (Figure 2B), it is possible that the activity detected in the 'normal' breast tissue adjacent to the tumour in specimen 313 represents unrecognized contamination of this tissue with cancer cells, or activation of the enzyme in response to the tumour. It is also possible that increased CaM kinase III activity is a characteristic of malignant precursors. In fact, we have detected low CaM kinase III activity in specimens from patients with pure ductal carcinoma in situ (DCIS) (TJ Kearney, TG Palmer, VH Vyas and WN Hait unpublished data). Histological examination determined that the cellularity of the tumour specimens was greater than that of the adjacent normal tissue. Therefore, we reassayed five representative specimens normalizing for cell number. The activity of CaM kinase III, whether normalized for protein or cell number, was markedly increased in breast tumour specimens compared with that of normal adjacent breast tissue.

An alternative explanation for the apparent increase in CaM kinase III activity in breast carcinoma was the presence of endogenous inhibitors of eEF-2 phosphorylation in normal mammary tissue. This was excluded by mixing equivalent amounts of homogenates from normal mammary tissue and breast tumours, in which we found no inhibition of eEF-2 phosphorylation (data not shown). We also determined whether the absence of eEF-2 phosphorylation in normal mammary tissue was due to the presence of increased phosphatase activity. Purified eEF-2 was phosphorylated by purified CaM kinase III in the presence of [$\gamma$-$^{32}$P]ATP followed by the addition of 10 mM ATP. This [$^{32}$P]eEF-2 was then incubated with homogenates of normal mammary gland for 0–20 min. No degradation of added [$^{32}$P]eEF-2 was observed during this incubation period (data not shown).

These data may have implications for the diagnosis of breast cancer. CaM kinase III activity was virtually absent in normal breast tissue. When detected in normal tissue adjacent to the breast cancer, the normal tissue was found to contain infiltrating tumour cells. Therefore, detection of CaM kinase III activity may be an early marker of invasive breast cancer.

The activity of CaM kinase III is stimulated by peptide growth factors. As shown in Figure 3A, serum deprivation significantly

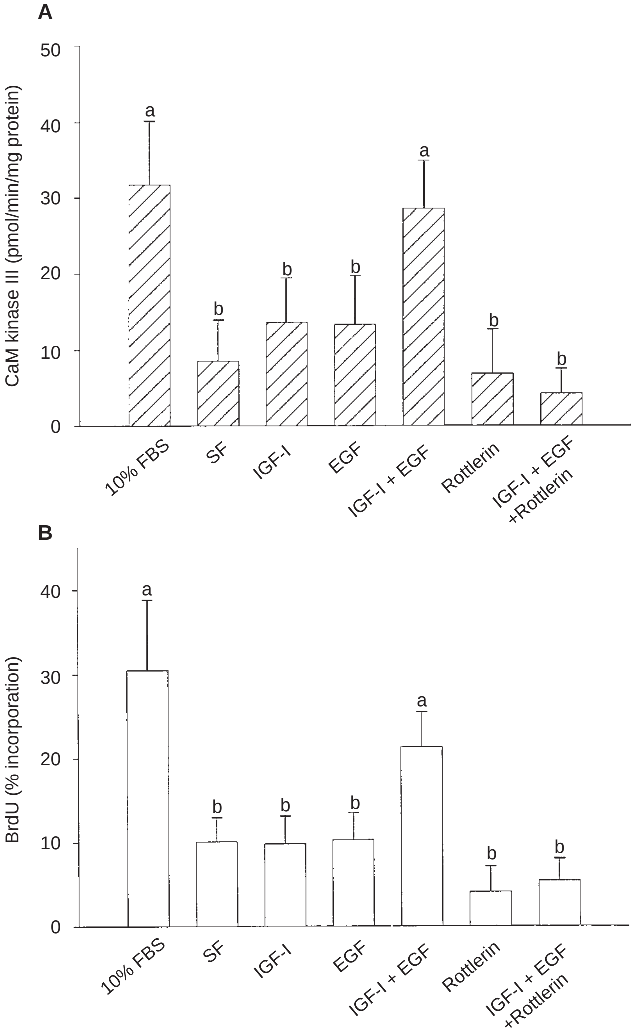

**Figure 3** Effect of growth factors and rottlerin on CaM kinase III activity and cell proliferation in MDA-MB-231 breast cancer cells. Cells in logarithmic phase were plated initially in serum-supplemented media as described in Materials and methods. After a 4–6-h attachment period, cells were washed with serum-free media (MCDB 105) and cultured for 24 h without mitogens. Cells were then exposed to either EGF (10 ng ml$^{-1}$) and/or IGF-I (20 ng ml$^{-1}$), rottlerin (5 $\mu$M), or 10% FBS for 24 h. Control cells were maintained in serum-free media without growth factors. (**A**) CaM kinase III activity was measured in cell lysates as described in Figure 1. Each bar represents the mean ± s.e.m. from three separate experiments ($P \leq 0.05$, a vs b). (**B**) Cell proliferation was measured by BrdU incorporation as described in Materials and methods. Fluorescence intensities were determined by quantitative flow cytometry and profiles were generated on a Becton Dickinson FACScan analyser. Each bar represents the mean ± s.e.m. from three separate experiments ($P \leq 0.10$, a vs b)

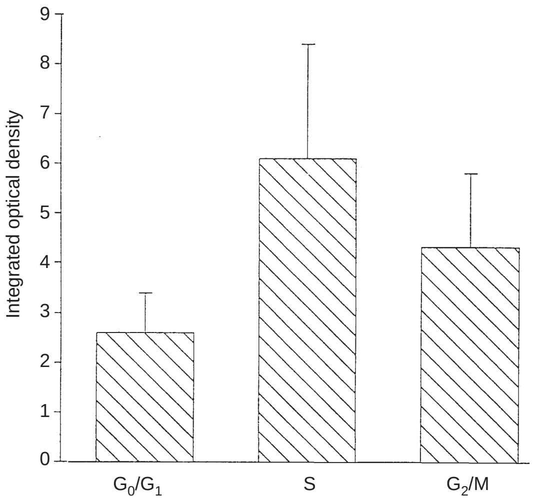

**Figure 4** CaM kinase III activity during the cell cycle. MCF-7 cells were plated at 1 × 10$^{5}$ cells ml$^{-1}$. At 48 h after plating, cells were trypsinized and separated by fluorescence-activated cell sorting as described in Materials and methods. Equal amounts of protein or equal cell numbers from each fraction were assayed for CaM kinase III activity as described in Figure 1. Each bar represents the mean ± s.e.m. for each phase of the cell cycle from three separate experiments

© Cancer Research Campaign 1999

decreased ($P \leq 0.05$) CaM kinase III activity and cell proliferation in MDA-MB-231 cells to 13% of that seen when cells were maintained in 10% FBS. Treatment of serum-deprived MDA-MB-231 cells with either IGF-I or EGF alone stimulated CaM kinase III activity to ≤ 35% of that of 10% serum (Figure 3B). In contrast, the combination of these two growth factors activated CaM kinase III levels to that observed in the presence of 10% serum ($P \leq 0.05$) (Figure 3A). Whereas the individual growth factors failed to stimulate cell proliferation, the combination of IGF-I and EGF significantly increased proliferation to levels similar to that observed in cells maintained in 10% serum ($P \leq 0.10$) (Figure 3B). The activity of CaM kinase III and cell proliferation induced by EGF and IGF-I was blocked by rottlerin, a CaM kinase III inhibitor (Figure 3). MDA-MB-231, an oestrogen-independent breast cancer cell line, was used to isolate the effects of peptide growth factors from that of steroid hormones on CaM kinase III activity. We are currently conducting experiments using MCF-7 cells to determine whether oestrogen can regulate the activity of this enzyme. Preliminary data (TJ Kearney et al, unpublished data) suggest that oestradiol also stimulates CaM kinase III activity in breast cancer cells.

We and others obtained similar results for CaM kinase III in other tissues. For example, Bagaglio et al (1994) found that serum deprivation markedly diminished the expression of CaM kinase III activity in rat glioma cells, whereas addition of serum promoted the onset of proliferation and the activation of the kinase. Other growth factors that activate CaM kinase III have been reported to stimulate the growth of fibroblasts such as EGF, vasopressin, bradykinin (Palfrey et al, 1987) and insulin (Levenson and Blackshear, 1989).

Previous studies have shown that rottlerin, a 5,7-dihydroxy-2,2-dimethyl-6-(2,4,6-trihydroxy-3-methyl-5-acetylbenzyl)-8-cinnamoyl-1,2-chromene isolated from the pericarps of *Mallotus phillipinensis* (Gschwendt et al, 1994), was an effective inhibitor of CaM kinase III activity in glioma cells (Parmer et al, 1997). Rottlerin blocks growth factor induced activation of CaM kinase III and blocks cell proliferation in breast cancer cells (Figure 3). Rottlerin has also been reported to inhibit the activity of PKC-δ. However, it was shown that it was the activation of PKC-δ that led to growth inhibition in NIH 3T3 cells (Mischak et al, 1993) and cell cycle arrest in CHO cells (Watanabe et al, 1992). The signal transduction cascade and proliferative response initiated by IGF-I and EGF in breast cancer activates CaM kinase III and inhibition of this enzyme, by rottlerin, blocks growth factor-induced proliferation. Therefore, the identification of more potent and selective CaM kinase III inhibitors may help identify new breast cancer therapies.

The response to growth factors requires a complex interaction between growth factors and growth factor receptors, followed by the activation of well-defined signal transduction pathways that mediate a proliferative response. This area of biology has contributed to our modern understanding of cellular proliferation and to the development of new targets for anti-cancer therapies. In breast cancer, this understanding led to the development of anti-oestrogens such as tamoxifen. Although much recent attention has focused on growth factor/receptor interactions, such as the immediate downstream effector elements seen on receptor-activated tyrosine kinases, less attention has been given to signalling events further downstream. In this regard, CaM-dependent pathways represent a compelling area for investigation into the proliferative responses of malignant cells. Numerous studies have suggested the importance of CaM-mediated signalling in cell proliferation and malignant transformation (Chafouleas et al, 1981; MacManus et al, 1981; Veigl et al, 1984; Rasmussen and Means, 1987, 1989; Lu and Means, 1993). Early studies from our laboratory and others detected increased CaM in malignant cells (Hait and DeRosa, 1988; Rasmussen and Means, 1989; Lu and Means, 1993), and demonstrated that drugs that bind directly to CaM and inhibit its function were both antiproliferative and cytotoxic to breast cancer cells (Hait and Lee, 1985; Ford et al, 1989). Studies by Means and colleagues demonstrated that overexpression of a CaM minigene increased proliferation, and we and others found CaM antisense to be antiproliferative and cytotoxic (Rasmussen and Means, 1989; Prostko et al, 1997).

CaM kinase III may also provide important new insights into the molecular coordination required for cell proliferation because this enzyme transiently inhibits protein synthesis through the phosphorylation of eEF-2 (Ryazanov and Spirin, 1993). The activation of CaM kinase III and subsequent inhibition of protein synthesis may help to explain a variety of experimental observations; transient inhibition of protein synthesis is an early event in mitogenesis (Fan and Penman, 1970; Celis et al, 1990). Furthermore, inhibitors of protein synthesis such as cycloheximide and anisomycin mimic many effects of growth factors, including the stimulation of cells into S-phase (Muller et al, 1984; Greenberg et al, 1986). The requirement for this effect is still unknown, but may represent a cellular mechanism for the degradation of short-lived cell cycle repressors (Ryazanov and Spirin, 1993). Thus, the rapid activation of CaM kinase III activity by mitogens may help explain the mechanism by which the cell may release itself from a cell cycle checkpoint.

To determine the stage of the cell cycle in which CaM kinase III activity was expressed, non-synchronized populations of MCF-7 cells were sorted and analysed. Figure 4 demonstrates that the activity of CaM kinase III per cell increased two- to threefold in non-synchronized S-phase cells. The data are similar to those of Carlberg et al (1991), who also found that CaM kinase III was increased during the S-phase of the cell cycle in Ehrlich ascites cells. Okmura-Noji and co-workers (1990) found that glial maturation factor increased the activity of CaM kinase III in glioblasts and glioblastoma cells as they entered the S-phase, and that this increase was blocked by a CaM antagonist.

In summary, these studies demonstrate that CaM kinase III activity is markedly elevated in human breast cancer. Furthermore, the activity of the enzyme is regulated by growth factors, and drugs that inhibit CaM kinase III activity block cell proliferation. These data suggest that aberrant CaM-dependent cell signalling pathways exist in human breast cancer and that CaM kinase III may represent a new target for novel anti-cancer therapies.

### ACKNOWLEDGEMENTS

The authors wish to thank Adam Cornfield and Nishita Desai of the Tissue Retrieval Service of The Cancer Institute of New Jersey, and Dr Alexy Ryazanov, Department of Pharmacology/UMDNJ for helpful comments.

### REFERENCES

Bagaglio DM and Hait WN (1994) Role of calmodulin-dependent phosphorylation of elongation factor 2 in the proliferation of rat glial cells. *Cell Growth Differ* **5**: 1403–1408

Carlberg U, Nilsson A, Skog S, Palmquist K and Nygard O (1991) Increased activity of the eEF-2 specific, $Ca^{2+}$ and calmodulin dependent protein kinase III during the S-phase in Ehrlich ascites cells. *Biochem Biophys Res Commun* **180**: 13472–13474

© Cancer Research Campaign 1999

Celis JE, Madsen P and Ryazanov AG (1990) Increased phosphorylation of elongation factor 2 during mitosis in transformed human amnion cells correlates with a decreased rate of protein synthesis. *Proc Natl Acad Sci USA* **87**: 4231–4235

Chafouleas JG, Pardue RLM, Brinkley BR, Dedman JR and Means AR (1981) Regulation of intracellular levels of calmodulin and tubulin in normal and transformed cells. *Proc Natl Acad Sci USA* **78**: 996–1000

Cheng EHC, Gorelick FS, Czernik AJ and Hait WN (1995) Calcium/calmodulin-dependent binding proteins and kinase activity in murine and human glioblastoma. *Cell Growth Differ* **6**: 615–621

Fan H and Penman S (1970) Regulation of protein synthesis in mammalian cells: inhibition of protein synthesis at the level of initiation during mitosis. *J Mol Biol* **50**: 655–656

Ford JM, Prozialeck WC and Hait WN (1989) Structural features determining activity of phenothiazines and related drugs for inhibition of cell growth and reversal of multidrug resistance. *Mol Pharmacol* **35**: 105–115

Futey LM, Medley QG, Cote GP and Egelhoff TT (1995) Structural analysis of myosin heavy chain kinase A from *Dictyostelium. J Biol Chem* **270**: 523–529

Greenberg ME, Hermanowski AL and Ziff EB (1986) Effect of protein synthesis inhibitors on growth factor activation of *c-fos*, *c-myc*, and actin gene transcription. *Mol Cell Biol* **6**: 1050–1057

Gschwendt M, Muller H-J, Kielbassa K, Zang R, Kittstein W, Rincke G and Marks F (1994) Rottlerin, a novel protein kinase inhibitor. *Biochem Biophys Res Commun* **199**: 93–98

Hait WN and Lee GL (1985) Characterization of the cytotoxic effects of calmodulin inhibitors. *Biochem Pharmacol* **34**: 3973–3978

Hait WN and Lazo JS (1986) Calmodulin: a potential target for cancer chemotherapeutic agents. *J Clin Oncol* **4**: 994–1012

Hait WN and DeRosa WT (1988) Calmodulin as a target for chemotherapeutic strategies. *Cancer Invest* **6**: 499–511

Hait WN, Ward MD, Trakht IN and Ryazanov AG (1996) Elongation factor-2 kinase: immunological evidence for the existence of tissue-specific isoforms. *FEBS Lett* **397**: 55–60

Levenson RM and Blackshear PJ (1989) Insulin-stimulated protein tyrosine phosphorylation in intact cells evaluated by giant two-dimensional gel electrophoresis. *J Biol Chem* **264**: 19984–19993

Lu KP and Means AR (1993) Regulation of the cell cycle by calcium and calmodulin. *Endocrine Rev* **14**: 40–58

MacManus JP, Braceland BM and Rixon RH (1981) An increase in calmodulin during growth of normal and cancerous liver in vivo. *FEBS Lett* **133**: 99–102

Makino R, Hayashi K and Sugimura T (1984) c-myc transcript is induced in rat liver at a very early stage of regeneration by cycloheximide treatment. *Nature* **310**: 697–698

Mischak H, Goodnight J, Kolch W, Martiny-Baron G, Schaechtle C, Kazanietz MG, Blumberg PM, Pierce JH and Mushinski JF (1993) Overexpression of protein kinase C-δ and -ε in NIH 3T3 cells induces opposite effects on growth, morphology, anchorage dependence, and tumorigenicity. *J Biol Chem* **268**: 6090–6096

Muller R, Bravo R, Burkhardt J and Curran T (1984) Induction of *c-fos* gene and protein by growth factors precedes activation of *c-myc*. *Nature* **312**: 716–720

Okumura-Noji K, Kato T, Ito J, Suzuki T and Tanaka R (1990) Stimulation by glial maturation factor of $Ca^{2+}$-dependent phosphorylation of $M_r$ 100 protein in rat glioblasts. *Neurochem Int* **17**: 559–571

Palfrey HC, Nairn AC, Muldoon L and Villereal ML (1987) Rapid activation of calmodulin-dependent protein kinase III in mitogen-stimulated human fibroblasts. *J Biol Chem* **262**: 9785–9792

Parmer TG, Ward MD and Hait WN (1997) Effects of rottlerin, an inhibitor of calmodulin-dependent protein kinase III, on cellular proliferation, viability, and cell cycle distribution in malignant glioma cells. *Cell Growth Differ* **8**: 327–334

Prostko CR, Zhang C and Hait WN (1997) The effect of altered cellular calmodulin expression on the growth and viability of C6 glioblastoma cells. *Oncology Res* **9**: 13–17

Rasmussen CD and Means AR (1987) Calmodulin is involved in regulation of cell proliferation. *EMBO J* **6**: 3961–3968

Rasmussen CD and Means AR (1989) Calmodulin is required for cell-cycle progression during $G_1$ and mitosis. *EMBO J* **8**: 73–82

Redpath NT, Price NT and Proud CG (1996) Cloning and expression of cDNA encoding protein synthesis elongation factor-2 kinase. *J Biol Chem* **271**: 17547–17554

Ryazanov AG and Spirin AS (1993) Phosphorylation of elongation factor 2. A mechanism to shut off protein synthesis for reprogramming gene expression. In *Translational Regulation of Gene Expression 2*. Ilan J (ed.), pp. 433–455. Plenum Press: NY, USA

Ryazanov AG, Ward MD, Mendola CE, Pavur KS, Dorovkov MV, Wiedmann M, Erdjument-Bromage H, Tempst P, Parmer TG, Prostko CR, Germino FJ and Hait WN (1997) Identification of a new class of protein kinases represented by eukaryotic elongation factor 2 kinase. *Proc Natl Acad Sci USA* **94**: 4884–4889

Snedecor GW and Cochran WG (1980) *Statistical Methods*. Iowa State University Press: Iowa, USA

Veigl ML, Vanaman TC, Brance ME and Sedwick WD (1984) Differences in calmodulin levels of normal and transformed cells as determined by culture conditions. *Cancer Res* **44**: 3184–3189

Watanabe T, Ono Y, Taniyama Y, Hazama K, Igarashi K, Ogita K, Kikkawa U and Nishizuka Y (1992) Cell division arrest induced by phorbol ester in CHO cells overexpressing protein kinase C-δ subspecies. *Proc Natl Acad Sci USA* **89**: 10159–10163

 © Cancer Research Campaign 1999

## References

[PDF_00544] Bagaglio D. M., Hait W. N. (1994). Role of calmodulin-dependent phosphorylation of elongation factor 2 in the proliferation of rat glial cells.. Cell Growth Differ.

[PDF_00553] Celis J. E., Madsen P., Ryazanov A. G. (1990). Increased phosphorylation of elongation factor 2 during mitosis in transformed human amnion cells correlates with a decreased rate of protein synthesis.. Proc Natl Acad Sci U S A.

[PDF_00557] Chafouleas J. G., Pardue R. L., Brinkley B. R., Dedman J. R., Means A. R. (1981). Regulation of intracellular levels of calmodulin and tubulin in normal and transformed cells.. Proc Natl Acad Sci U S A.

[PDF_00560] Cheng E. H., Gorelick F. S., Czernik A. J., Bagaglio D. M., Hait W. N. (1995). Calmodulin-dependent protein kinases in rat glioblastoma.. Cell Growth Differ.

[PDF_00563] Fan H., Penman S. (1970). Regulation of protein synthesis in mammalian cells. II. Inhibition of protein synthesis at the level of initiation during mitosis.. J Mol Biol.

[PDF_00566] Ford J. M., Prozialeck W. C., Hait W. N. (1989). Structural features determining activity of phenothiazines and related drugs for inhibition of cell growth and reversal of multidrug resistance.. Mol Pharmacol.

[PDF_00569] Futey L. M., Medley Q. G., Côté G. P., Egelhoff T. T. (1995). Structural analysis of myosin heavy chain kinase A from Dictyostelium. Evidence for a highly divergent protein kinase domain, an amino-terminal coiled-coil domain, and a domain homologous to the beta-subunit of heterotrimeric G proteins.. J Biol Chem.

[PDF_00571] Greenberg M. E., Hermanowski A. L., Ziff E. B. (1986). Effect of protein synthesis inhibitors on growth factor activation of c-fos, c-myc, and actin gene transcription.. Mol Cell Biol.

[PDF_00574] Gschwendt M., Müller H. J., Kielbassa K., Zang R., Kittstein W., Rincke G., Marks F. (1994). Rottlerin, a novel protein kinase inhibitor.. Biochem Biophys Res Commun.

[PDF_00581] Hait W. N., DeRosa W. T. (1988). Calmodulin as a target for new chemotherapeutic strategies.. Cancer Invest.

[PDF_00579] Hait W. N., Lazo J. S. (1986). Calmodulin: a potential target for cancer chemotherapeutic agents.. J Clin Oncol.

[PDF_00577] Hait W. N., Lee G. L. (1985). Characteristics of the cytotoxic effects of the phenothiazine class of calmodulin antagonists.. Biochem Pharmacol.

[PDF_00583] Hait W. N., Ward M. D., Trakht I. N., Ryazanov A. G. (1996). Elongation factor-2 kinase: immunological evidence for the existence of tissue-specific isoforms.. FEBS Lett.

[PDF_00586] Levenson R. M., Blackshear P. J. (1989). Insulin-stimulated protein tyrosine phosphorylation in intact cells evaluated by giant two-dimensional gel electrophoresis.. J Biol Chem.

[PDF_00589] Lu K. P., Means A. R. (1993). Regulation of the cell cycle by calcium and calmodulin.. Endocr Rev.

[PDF_00591] MacManus J. P., Braceland B. M., Rixon R. H., Whitfield J. F., Morris H. P. (1981). An increase in calmodulin during growth of normal and cancerous liver in vivo.. FEBS Lett.

[PDF_00593] Makino R., Hayashi K., Sugimura T. (1984). C-myc transcript is induced in rat liver at a very early stage of regeneration or by cycloheximide treatment.. Nature.

[PDF_00596] Mischak H., Goodnight J. A., Kolch W., Martiny-Baron G., Schaechtle C., Kazanietz M. G., Blumberg P. M., Pierce J. H., Mushinski J. F. (1993). Overexpression of protein kinase C-delta and -epsilon in NIH 3T3 cells induces opposite effects on growth, morphology, anchorage dependence, and tumorigenicity.. J Biol Chem.

[PDF_00601] Müller R., Bravo R., Burckhardt J., Curran T. (1984). Induction of c-fos gene and protein by growth factors precedes activation of c-myc.. Nature.

[PDF_00607] Palfrey H. C., Nairn A. C., Muldoon L. L., Villereal M. L. (1987). Rapid activation of calmodulin-dependent protein kinase III in mitogen-stimulated human fibroblasts. Correlation with intracellular Ca2+ transients.. J Biol Chem.

[PDF_00610] Parmer T. G., Ward M. D., Hait W. N. (1997). Effects of rottlerin, an inhibitor of calmodulin-dependent protein kinase III, on cellular proliferation, viability, and cell cycle distribution in malignant glioma cells.. Cell Growth Differ.

[PDF_00613] Prostko C. R., Zhang C., Hait W. N. (1997). The effects of altered cellular calmodulin expression on the growth and viability of C6 glioblastoma cells.. Oncol Res.

[PDF_00616] Rasmussen C. D., Means A. R. (1987). Calmodulin is involved in regulation of cell proliferation.. EMBO J.

[PDF_00618] Rasmussen C. D., Means A. R. (1989). Calmodulin is required for cell-cycle progression during G1 and mitosis.. EMBO J.

[PDF_00621] Redpath N. T., Price N. T., Proud C. G. (1996). Cloning and expression of cDNA encoding protein synthesis elongation factor-2 kinase.. J Biol Chem.

[PDF_00628] Ryazanov A. G., Ward M. D., Mendola C. E., Pavur K. S., Dorovkov M. V., Wiedmann M., Erdjument-Bromage H., Tempst P., Parmer T. G., Prostko C. R. (1997). Identification of a new class of protein kinases represented by eukaryotic elongation factor-2 kinase.. Proc Natl Acad Sci U S A.

[PDF_00637] Watanabe T., Ono Y., Taniyama Y., Hazama K., Igarashi K., Ogita K., Kikkawa U., Nishizuka Y. (1992). Cell division arrest induced by phorbol ester in CHO cells overexpressing protein kinase C-delta subspecies.. Proc Natl Acad Sci U S A.

